# Coalitionality shapes moral elevation: evidence from the U.S. Black Lives Matter protest and counter-protest movements

**DOI:** 10.1098/rsos.220990

**Published:** 2023-03-29

**Authors:** Colin Holbrook, Daniel M. T. Fessler, Adam Maxwell Sparks, Devin L. Johnson, Theodore Samore, Lawrence I. Reed

**Affiliations:** ^1^ Department of Cognitive and Information Sciences, University of California, Merced, CA 95343, USA; ^2^ Department of Anthropology, University of California, Los Angeles, CA 90095, USA; ^3^ Kindness Institute, University of California, Los Angeles, CA 90095, USA; ^4^ Center for Behavior, Evolution, and Culture, University of California, Los Angeles, CA 90095, USA; ^5^ Department of Psychology, University of Guelph, ON N1G1Y4, Canada; ^6^ Department of Psychology, Neuroscience and Behaviour, McMaster University, ON, Canada L8S4K1; ^7^ Department of Psychology, New York University, ON NY10003, USA

**Keywords:** prosociality, emotion, elevation, coalitional psychology, political orientation

## Abstract

Witnessing altruistic behaviour can elicit *moral elevation*, an emotion that motivates prosocial cooperation. This emotion is evoked more strongly when the observer anticipates that other people will be reciprocally cooperative. Coalitionality should therefore moderate feelings of elevation, as whether the observer shares the coalitional affiliation of those observed should influence the observer's assessment of the likelihood that the latter will cooperate with the observer. We examined this thesis in studies contemporaneous with the 2020 Black Lives Matter (BLM) protests. Although BLM protests were predominantly peaceful, they were depicted by conservative media as destructive and antisocial. In two large-scale, pre-registered online studies (total *N* = 2172), political orientation strongly moderated feelings of state elevation elicited by a video of a peaceful BLM protest (Studies 1 and 2) or a peaceful Back the Blue (BtB) counter-protest (Study 2). Political conservatism predicted less elevation following the BLM video and more elevation following the BtB video. Elevation elicited by the BLM video correlated with preferences to defund police, whereas elevation elicited by the BtB video correlated with preferences to increase police funding. These findings extend prior work on elevation into the area of prosocial cooperation in the context of coalitional conflict.

## 1. Introduction

From warfare to sports to political contests, the emotions aroused by collective striving, triumph or defeat depend on the side one favours. Here, we examine the role of partisan attitudes in shaping emotional responses within the context of a deeply moralized, politically divisive, ongoing societal conflict: the Movement for Black Lives in the United States [1]. Although various particulars of this conflict are unique to the present historical moment, the functional logic of in-group cooperation can inform understanding of emotional reactions to this and other conflicts.

The various experiences commonly described as ‘emotions’ can be understood as the product of corresponding adaptations produced by natural selection. Emotion adaptations are composed of transitory entrainments of diverse perceptual, somatic and cognitive mechanisms that generate responses which, on average, effectively addressed recurring challenges and opportunities in the social and physical lives of ancestral hominids over evolutionary time [2–4]. For example, ‘anger’ broadly concerns scenarios in which agents have thwarted the fulfillment of one's preferences, marshalling thoughts, feelings and behaviours directed toward punishing—and thereby deterring—transgressors. Crucially, such evolved emotional responses are theorized to be contextually sensitive to moderating factors [5], including past interactions with transgressors, the nature of their relationship with the self (e.g. kin, coalitional ally, romantic partner and local status-holder), what social or material resources they may have at their disposal relative to the self, what local norms govern societally appropriate expressions of punishment, and so on [6,7]. From a proximate neural perspective, emotions should be expected to display such context-sensitive variation because they are in part composed of cortical mechanisms related to behavioural flexibility and learning [8,9]. From an ultimate functional perspective, given that natural selection favours adaptive contextual variability, we should observe strategic modulations of emotional responses that align with fitness incentives [5].

The Attitude-Scenario-Emotion (ASE) framework models the process by which representations acquired through experience moderate emotional reactions in a circumstantially contingent, fitness-enhancing way [10]. In the ASE model, what are termed attitudes are encoded expectations regarding the relative value and likely behaviour of others—what will they do, and how will this impact one's personal welfare and preferences? In this way, attitudes shape appraisals of social scenarios as they arise, determining the elicitation of emotional responses that vary depending on the persons involved. Returning to anger as an illustration, scenarios involving transgressive harm to the self [11] or to kin [12] evoke greater feelings of anger, and motivation to directly confront perpetrators, than do scenarios involving harm to acquaintances, which evoke relatively greater feelings of disgust associated with tendencies to withdraw from perpetrators. These patterns are in line with divergent fitness incentives to risk versus avoid aggressive conflict contingent on the identity of the victim. In other words, the emotions and related behaviours elicited depend on the perceived stakes of the transgression scenario, and these stakes are a function of attitudes toward the victim as more or less valuable to the self. Likewise, anger and related inclinations toward punishment are moderated by the identity of the transgressor, such that kin or friends who inflict harm elicit both relatively muted feelings of anger and heightened inclinations to forgive [13]. Put simply, how one feels in response to an event critically hinges on one's attitude toward those involved.

Just as attitudes toward particular individuals adaptively structure emotional responses, attitudes regarding the social milieu in which events occur should also be expected to influence scenario appraisals and related emotional responses. In the case of *moral elevation,* an emotion designed to heighten prosociality upon witnessing the exceptionally prosocial behaviour of others [14,15], baseline attitudinal expectations of the cooperativeness of other people moderate the degree to which individuals experience elevation [16]. The effect of attitudes regarding prevailing levels of prosociality on experiences of elevation makes adaptive sense, given that the profitability of prosociality is contingent on the responses of others in one's social environment [17]. Individuals embedded in social environments in which they have experienced low levels of cooperation benefit less from engaging in prosocial behaviour, as the costs incurred by helping would generally not be outweighed by direct or indirect reciprocity from others, or other downstream benefits related to prosociality [18]. Reflecting this functional logic, individuals who regard their communities as non-cooperative appear to appraise helping scenarios as less representative of their own social milieu, or even, at an extreme, as cynical or deceitful ploys, thus muting the elicitation of elevation [16]. Conversely, in cooperative social contexts that engender idealistic attitudes regarding the cooperativeness of others, upregulating one's prosocial inclinations in response to cues of others' helpfulness yields significant fitness benefits (i.e. through direct or indirect reciprocity, reputation enhancement and/or inclusion in cooperative endeavours). Correspondingly, when individuals who harbour the expectation that others in their community are cooperative witness extraordinary acts of prosociality, they experience relatively high levels of state elevation and increases in the desire to engage in cooperative behaviour [16,19]. Prior work on elevation has predominantly focused on prosociality within broadly shared communities under circumstances lacking salient conflict [15,20–22]. However, cooperation often occurs in the context of conflict between coalitions. Here, we focus on elevation and prosociality in the context of such conflict.

## 2. Coalitionality and cooperation

Coalitionality, like elevation, appears rooted in the need for individuals to reap the advantages of cooperation while avoiding exploitation [23]. Providing resources to others can yield benefits via reciprocity, but also renders one vulnerable. Ascertaining who shares a positive investment in a common in-group, appraising these individuals as more valuable and helpful than members of out-groups, and cooperating accordingly, can increase the benefits that the individual obtains while also enhancing group coordination [24–26]. In this way, the psychological mechanisms enabling assortment and cooperation on the basis of group identity resemble the ASE processes of elevation: representations of the likely cooperativeness of others toward oneself—indexed by their coalitional affiliations—shape appraisals of events concerning them, and motivate responses accordingly. Synthesizing the logic of coalitionality with the ASE model of elevation, the prosociality of out-group members toward one another should elicit less elevation than the same acts when conducted by in-group members, because the latter hold greater potential to yield fitness benefits should the observer increase their own prosocial behaviour. Consistent with this premise, an extensive empirical literature documents that individuals are indeed more willing to cooperate with in-group members than with out-group members [27,28]; at the proximate level, witnessing out-group members suffering physical pain arouses less activation of neural regions linked with empathy than does equivalent observations of in-group members [29,30].

Active intergroup conflict further incentivizes cooperation between in-group members [31,32]. Cooperation during periods of conflict strengthens social alliances that may be particularly vital under conflict-related contexts of danger or deprivation [33] and also increases the group's competitive ability, thus enhancing both individual and group-level fitness [34–36]. Correspondingly, motivation to reward in-group cooperators as well as punish non-cooperators has been observed to increase during violent intergroup conflict [35], and a cross-cultural meta-analysis indicates that exposure to warfare increases subsequent prosociality in dealings with in-group members, but not out-group members [37]. In addition to such real-world evidence, numerous studies—predominantly drawing on priming and/or counterfactual methods—report that individuals parochially favour in-group members and/or derogate out-group members to a greater extent following cues of various threats [38–40]. Likewise, a laboratory manipulation of group conflict, contrasted with within-group framings, of team Prisoner's Dilemma games found greater monetary contributions (i.e. cooperation) when the game involved intergroup conflict [41]. In laboratory paradigms modelling intergroup conflict, studies of individual differences reveal that highly prosocial individuals are more inclined to sacrifice in order to aid their own coalitions, but not out-groups [42,43]. Taken together, the foregoing literatures converge to predict that group identification will moderate feelings of elevation elicited by cues of cooperation evinced, respectively, by members of the in-group or the out-group, and that this will be particularly true in contexts of intergroup conflict.

## 3. Partisan political attitudes and the Black Lives Matter movement

Originally coalescing in 2013 following the acquittal of the man who fatally shot unarmed teenager Trayvon Martin [44], the Black Lives Matter (BLM) movement grew in response to a number of other widely publicized police killings of unarmed Black persons, then dramatically expanded in scale and public attention in the aftermath of the police murder of George Floyd in the spring of 2020. BLM is a decentralized political movement made up of an array of contributing organizations and individuals that, while somewhat heterogeneous regarding policy priorities or tactics [45,46], uniformly advocate for the reform of the police's treatment of Black people, notably including proposals to reallocate police funds to address social issues such as homelessness, educational inequities or mental health treatment [47,48]. While attracting an estimated tens of millions of U.S. protesters to the streets to demand racial justice in policing [49], the BLM protests also inspired counter-protests on behalf of organizations voicing support for police, variously described as Blue Lives Matter or Back the Blue (BtB) [50]. The latter organizations originally arose in the aftermath of protests in response to the fatal police shooting of unarmed teenager Michael Brown and police strangulation of Eric Garner in 2014 [51], following the killings of police officers Rafael Ramos and Wenjian Liu by an individual who purportedly sought vengeance for the deaths of Brown & Garner [51]. BtB protesters deny the existence of racial inequities in policing, reject calls for police reform and advocate for legislation classifying attacks on police officers as hate crimes [52]. Although far smaller in scale than BLM protests, BtB counter-protests during 2020 were estimated to have drawn thousands of supporters [53,54].

The majority of U.S. BLM protests in 2020 were peaceful, with property damage, looting or violence estimated to have occurred in less than 6% of protests [50], including destructive acts committed by opportunistic non-protesters. Further, within the relatively few protests in which injuries were documented, in many instances the violence was perpetrated by counter-protesters or police officers [55]. Nonetheless, politically conservative news media predominantly depicted the BLM protests as destructive and violent [56], and then-President Donald Trump, a Republican, called for the restoration of ‘law and order’, publicly described the BLM logo as a ‘symbol of hate’ [57], and labelled the protesters as far-left ‘thugs’ and ‘anarchists’ whose goals included ‘the destruction of the nuclear family, [as well as to] abolish the police, abolish prisons, abolish border security, abolish capitalism’ [58]. By contrast, prominent officials in the Democratic Party issued statements broadly supportive of the protests. For example, then-Speaker of the House Nancy Pelosi described the BLM movement as ‘peacefully protesting to demand an end to the pattern of racial injustice and police brutality that has killed so many innocent Americans' [59].

The politicized nature of the public discourse was reflected by divergent partisan perceptions of the BLM movement. Seventy-three per cent of Republicans polled in June of 2020 rated President Trump's statements about the BLM protests as either completely or mostly correct, whereas 91% of Democrats rated those statements as wrong [60]. Roughly 80% of Republicans and those who leaned Republican viewed the protests as an excuse to engage in criminal behaviour, compared with 40% of Democrats and Democrat-leaning respondents [60]. Conversely, only 45% of Republicans relative to 84% of Democrats viewed BLM protesters as motivated by genuine concerns about the unjust treatment of Black people. When polled three months later, a mere 20% of Republicans or Republican-leaning respondents reported supporting the BLM movement to any extent, whereas 88% of Democrats and those who leaned Democratic supported BLM [61].

Taken as a whole, the 2020 polling data indicate that politically conservative individuals broadly categorized the BLM movement as a threatening, adversarial coalition of leftist extremists, whereas BtB counter-protesters were likely to be viewed as a coalition of likeminded individuals uniting to support police in their efforts to preserve peace and security. Inversely mirroring this partisan perspective, politically left-leaning individuals appear to have broadly valorized BLM as a social justice movement of likeminded individuals uniting in opposition to racially disproportionate police violence, with the BtB counter-protesters seen as a retrogressive coalition acting in opposition to the just aims of BLM. In sum, because the BLM and BtB protests were understood as rooted in moralized violence-related conflict, with partisan political attitudes largely determining observers' appraisal of the side regarded as prosocial and in alignment with the coalitional identity of the observer, the large-scale BLM protests of 2020 provided a real-world opportunity to explore the coalitional nature of elevation under contexts of intergroup antagonism.

## 4. Overview of studies

In two studies conducted while large-scale BLM demonstrations were occurring across the USA, we explored associations between political orientation, elevation and support for the BLM protest movement. In much prior elevation research, participants' reactions to stimuli depicting unusually prosocial behaviour are compared to reactions to behaviour lacking a prosocial element. We adapted this design to our present focus on coalitionality by employing stimuli depicting harmonious, coordinated behaviour in the context of political demonstrations. In Study 1, participants viewed brief videos depicting either BLM protesters or neutral control individuals, then reported their state feelings of elevation and their preferences regarding increasing versus decreasing police funding. Study 2 used the same design, adding a third video depicting BtB protesters (see the electronic supplementary material for a description of measures used in a pilot study but not referenced further: self-reported engagement with the BLM protests and opinions regarding social issues orthogonal to racial justice and/or policing).

## 5. Predictions

The ASE framework, articulated with models of partisan cooperation under intergroup conflict and applied to the BLM movement, generates a constellation of related predictions:

1 Political orientation will moderate elevation in response to video stimuli depicting each protest movement.
  a. Liberal [conservative] attitudes will correlate with greater [less] elevation following a BLM protest video. (Tested in Studies 1 and 2)
  b. Liberal [conservative] attitudes will correlate with less [greater] elevation following a BtB protest video. (Tested in Study 2)
  c. Political orientation will not predict elevation in an apolitical, affectively neutral control video condition. (Tested in Studies 1 and 2)
2 Partisan elevation will track partisan police funding preferences.
  a. Elevation in the context of a BLM protest video will positively correlate with preferring to decrease police funding. (Tested in Studies 1 and 2)
  b. Elevation in the context of a BtB protest video will positively correlate with preferring to increase police funding. (Tested in Study 2)
  c. Elevation will not predict funding preferences in the neutral video condition. (Tested in Studies 1 and 2)

To confirm that our measure of political orientation-tracked attitudes toward the police and the BLM protest movement as intended, we also planned to check whether:

– Liberal [conservative] political attitudes track preferences to decrease [increase] police funding.
– Political attitudes track appraisals of the BLM movement, BtB movement and police as prospective prosocial cooperators.

## 6. Study 1

### 6.1. Methods

The pre-registrations, full materials and datasets for the studies reported here are publicly archived (see https://osf.io/kdeg6/). All studies were approved by the University of California, Los Angeles, Institutional Review Board and performed in accordance with guidelines governing research with human participants. Informed consent was obtained from all participants prior to beginning each study.

#### 6.1.1. Participants

Based on results from our prior work [16], as well as a pilot study conducted a few weeks earlier on 24 June 2020 (see electronic supplementary material), we targeted a sample size of 1000 (500 per condition), recruited via Amazon Mechanical Turk (500 + completed assignments, 99% approval, located in the USA) in exchange for U.S. $1.25. Participants were recruited on 23 July 2020. Data were prescreened for repeat participation, minimal completeness, answering ‘catch questions,’ watching the entire video (based on a page timer) and technical problems with video streaming reported by participants. The final sample consisted of 856 participants (43.4% female, *M_age_* = 39.4 years, s.d. = 12.6). 71.4% of the participants identified as White, 11.5% as Black, and 17.1% as Other. With respect to political party affiliation, 27.8% identified as Republicans, 47.5% identified as Democrats and 24.8% identified as Independents.

#### 6.1.2. Design

In order to confirm that political conservatism indexes attitudes viewing police as prosocial cooperative partners, participants first completed a face-valid self-report measure consisting of five items assessing perceptions of the degree of prosociality characteristic of police (e.g. ‘Police are considerate of the interests of people in my broader community;’ *α* = 0.95; for descriptives, see electronic supplementary material, table S1).

Next, in a between-subjects design, participants were randomly assigned to view a video depicting either BLM protests or a neutral control (footage of individuals walking in a crowded city). The BLM video was composited from several contemporaneous media reports, edited with the intent of highlighting (i) the general aim of the protesters to increase racial equity in policing, (ii) prosocial coordination among the protesters and (iii) expressions of overt anger regarding police violence. A melodic musical score was layered over this composite of footage to render it more cohesive and to cue participants that the video portrays the BLM movement in a positive light. In sum, the BLM video was intended to elicit state elevation among politically liberal participants.

Following the video manipulation, participants completed a self-report emotion measure, including a 15-item elevation scale (overall *α* = 0.97) previously developed by Sparks *et al*. [16]. This scale is composed of three subscales focused, respectively, on folk affect emotion terms (e.g. ‘inspired’, ‘uplifted’; *α* = 0.98), somatic symptoms (e.g. ‘tears in eyes’; *α* = 0.90) and prosocial behavioural motives (e.g. ‘want to be a better person’; *α* = 0.95), using 4-point Likert scales (0 = *Not at all*; 1 = *Slightly*; 2 = *Moderately*; 3 = *Strongly*). Results reported in the main text use the overall elevation score; see electronic supplementary material (electronic supplementary material, figures S1–S3) for parallel analyses focused on the emotion terms, somatic symptoms and behavioural tendencies subscales as the outcome measures.

Next, in random order, participants reported their preferences with regard to police funding:

‘There have been proposals to reduce funding for police departments in order to invest that money in other social services. There have also been proposals to increase funding for police departments in order to uphold the rule of law and preserve order. If you could choose, how much would you decrease or increase funding to police departments?’

Participants used a slider to indicate their preference on a linear scale from a decrease of 100% (i.e. reallocating all funding away from policing) to an increase of 100% (i.e. doubling police funding).

Finally, we measured political orientation as part of the demographic questions, using a modification of Dodd *et al*.'s [62] version of Wilson & Patterson's [63] issues index. Participants were asked to indicate whether they agree, disagree or are uncertain about various prominent issues in contemporary U.S. politics (e.g. abortion, tax rates and gun control) which were then composited (*α* = 0.89). Agreement was scored as +1, disagreement as −1 and uncertainty as 0; liberal items were reverse scored, hence increasing positive values reflect greater conservatism. Responses were averaged such that a score of 1 [−1] would indicate uniformly conservative [liberal] positions. We used political orientation as a measure of coalitional left- versus right-wing attitudes toward the BLM movement. Once the study was complete, participants were thanked, debriefed and compensated. (The studies also included exploratory measures not discussed here (e.g. self-reports of online engagement about the protest movement, perceptions of societal issues orthogonal to racial inequities in policing) which are intended for separate publication.)

## 7. Results

### 7.1. Political orientation indexes attitudes toward Black Lives Matter

As anticipated, and in support of the use of overall political orientation as an index of attitudes toward the BLM movement, political conservatism positively correlated both with preferences to increase police funding, *r*(854) = 0.62, *p* < 0.001, and with idealistic attitudes toward the police as trustworthy and prosocial, *r*(854) = 0.49, *p* < 0.001 (for descriptives, see electronic supplementary material, table S1; for correlations between political orientation, idealistic attitudes, funding preferences and state elevation, see electronic supplementary material, tables S2 and S3). In sum, political orientation does appear to capture overall perceptions of the BLM movement. (Parallel analyses using the police- and BLM-attitude measures in place of political orientation produce closely comparable patterns of association; see electronic supplementary material, figures S4 and S5). Follow-up tests did not reveal an interaction between political orientation and video condition on funding preference, *p* = 0.668.

### 7.2. Political orientation and state elevation

**Figure 1.**
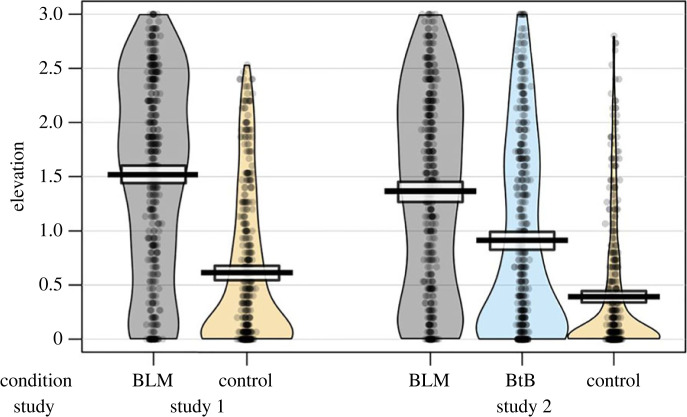
State elevation by video condition. BLM = Black Lives Matter; BtB = Back the Blue. Note that the neutral control videos used in Studies 1 and 2 differed.

**Figure 2.**
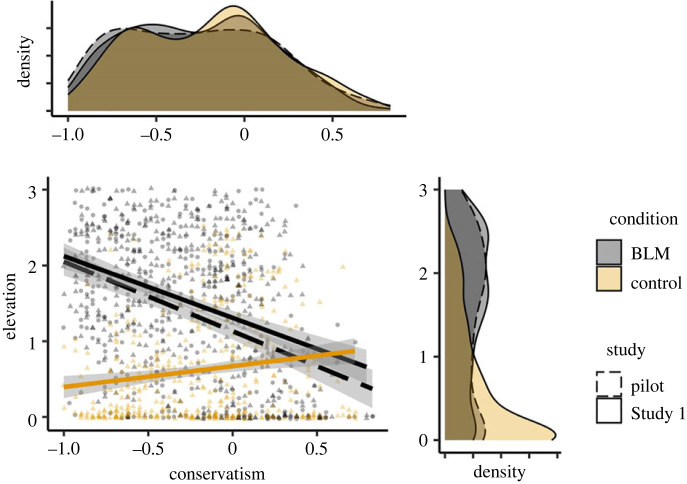
The association between political orientation and state elevation by video condition in the pilot study and Study 1. BLM = Black Lives Matter.

The political orientation of the overall sample was left-leaning, *M* = −0.23, s.d. = 0.40. Consistent with this left-leaning tendency, an analysis of variance (ANOVA) revealed state elevation to be greater in the BLM video condition, *M* = 1.52, s.d. = 0.91, than in the Control condition, *M* = 0.62, s.d. = 0.91 ($\eta_{p}^{2}=0.23$, *F*_1,854_ = 261.90, *p* < 0.001), figure 1. A moderated linear regression model including state elevation as the dependent variable, political orientation as the independent variable and video condition as the potential moderator, revealed a significant interaction, *b* = −1.09, s.e. = 0.13, *t* = −8.25, *p* < 0.001. Simple slopes analyses confirmed that, in line with Prediction 1, conservative attitudes were negatively associated with elevation in the BLM condition, *b* = −0.82, s.e. = 0.09, *t* = −8.69, *p* < 0.001, 95% CI = [−1.00, −0.63], but not the Control condition (figure 2). Against expectations, conservative attitudes predicted greater elevation in the Control condition, *b* = 0.28, s.e. = 0.09, *t* = 2.96, *p* < 0.001, 95% CI = [0.09, 0.46]. (See electronic supplementary material, table S2 for comparable associations using non-parametric Spearman rank-order tests given left-skewed ratings of elevation in the control condition).

### 7.3. Partisan elevation and police funding preferences

**Figure 3.**
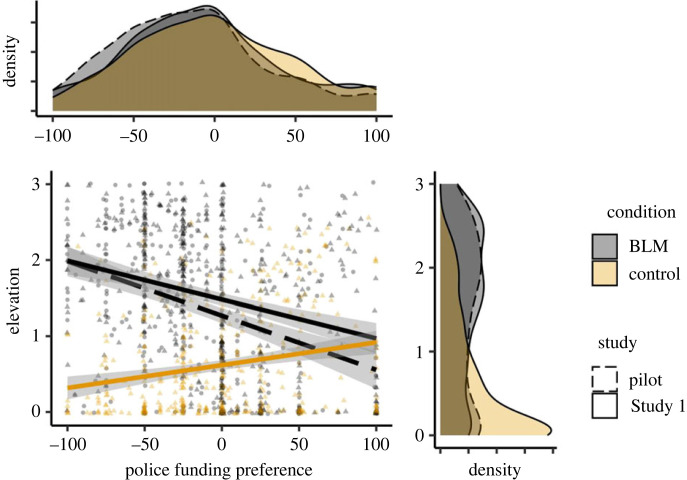
The association between police funding preferences and state elevation by video condition in the pilot study and Study 1. BLM = Black Lives Matter.

An ANOVA revealed a nonsignificant trend for participants to favour decreasing police funding in the BLM video condition, *M* = −7.07, s.d. = 48.21, to a greater extent than in the Control condition, *M* = −1.11, s.d. = 48.52 ($\eta_{p}^{2}=0.004$, *F*_1,854_ = 3.25, *p* = 0.072). We next tested the association between state elevation elicited by the BLM video manipulation and preferences to decrease versus increase funding for the police. A moderated linear regression model including police funding preferences as the dependent variable, state elevation as the independent variable, and video condition as the potential moderator, revealed a significant interaction, *b* = −28.48, s.e. = 4.08, *t* = −6.99, *p* < 0.001. Consistent with Prediction 2, simple slopes analyses confirmed that state elevation in the BLM video condition-tracked preferences to defund the police, *b* = −14.40, s.e. = 2.49, *t* = −5.77, *p* < 0.001, 95% CI = [−19.27, −9.48] (figure 3). In the Control video condition, against predictions, state elevation correlated with preferences to increase funding to the police, *b* = 14.10, s.e. = 3.22, *t* = 4.37, *p* < 0.001, 95% CI = [7.78, 20.44]. (See electronic supplementary material, table S2 for comparable associations using non-parametric Spearman rank-order tests given left-skewed ratings of elevation in the control condition.)

## 8. Discussion

In Study 1, we assessed the role of political orientation in moderating feelings of elevation elicited by a pro-BLM video relative to a neutral control video. As predicted, liberal [conservative] attitudes predicted significantly heightened [diminished] feelings of elevation in response to the BLM video relative to the control video, and feelings of state elevation engendered by the BLM video-tracked preferences to defund the police. This overall pattern supports the premise that partisan coalitional attitudes can moderate feelings of state elevation in response to cues of cooperation. However, the design of Study 1 failed to assess effects of cues of cooperation likely to elicit elevation among politically conservative individuals. Accordingly, in Study 2, we added a BtB video manipulation closely patterned after the BLM video created for Study 1.

Although the BLM video increased state elevation, there was no significant effect of the video condition, nor interaction between political orientation and video condition, on police funding preferences. These findings suggest that funding preferences reflected relatively stable political attitudes correlated with the propensity to experience coalitional elevation, rather than a decision regarding whether to behave prosocially that would theoretically be susceptible to the influence of state elevation.

Unexpectedly, political conservatism was modestly yet significantly positively correlated with state elevation in the control video condition. Speculatively, given the politicized nature of the COVID-19 pandemic in the USA [64] and the co-occurrence of lockdowns, social distancing, and mask mandates at the time of data collection, the control video's depiction of unmasked crowds interacting freely may have been perceived as elevating by conservatives, and/or as antisocial by liberals. Accordingly, we adopted a different control stimulus in Study 2 intended to minimize affective or political responses: an uneventful laptop repair video.

## 9. Study 2

### 9.1. Methods

#### 9.1.1. Participants

We targeted a sample size of 1500 (500 per condition), recruited via Amazon Mechanical Turk (500 + completed assignments, 99% approval, located in the USA) in exchange for $1.25. Participants were recruited on 18 December 2020. Data were prescreened as in Study 1, yielding a final sample consisting of 1316 participants (50.2% female, *M_age_* = 41.9 years, s.d. = 13.1). Of the participants, 73.2% identified as White, 9.0% as Black and 17.8% as Other; 24.0% identified as Republicans, 49.1% identified as Democrats and 26.9% identified as Independents.

#### 9.1.2. Design

As in Study 1, participants completed a self-report measure assessing perceptions of the degree of prosociality characteristic of police (*α* = 0.96), as well as a parallel measure added in Study 2 assessing perceptions of the BLM protesters (*α* = 0.94) (for descriptives, see electronic supplementary material, table S1). Next, in a between-subjects design, participants were randomly assigned to view a video depicting either BLM protests, BtB protests or a neutral control (footage of a laptop being repaired; see OSF). The BLM video was the same as used in Study 1. Paralleling the BLM video, the BtB video was composited from contemporaneous media reports, edited with the intent of highlighting (i) the general aim of the counter-protesters to refute the criticisms of the BLM protesters and support existing police institutions, (ii) prosocial coordination among the protesters and (iii) expressions of overt anger regarding the BLM protests. The same musical score was layered over the BtB footage as used in the BLM video, and the BtB sequence was patterned closely after the BLM video (see OSF). In sum, just as the BLM video was intended to elicit state elevation in politically liberal participants, the BtB video was intended to elicit state elevation in politically conservative participants.

Following the video manipulation, participants again completed the self-report 15-item elevation scale (overall *α* = 0.97; folk affect emotion terms subscale *α* = 0.98, somatic symptoms subscale *α* = 0.92, prosocial behavioural motives subscale *α* = 0.95; Sparks *et al*. [16]). Participants again reported their preferences with regard to police funding, then the modified political issues index (*α* = 0.89) [62]. Upon completing the study, participants were thanked, compensated and debriefed.

## 10. Results

### 10.1. Political orientation indexes attitudes toward Black Lives Matter

As in Study 1, political conservatism positively correlated with preferences to increase police funding, *r*(1,314) = 0.65, *p* < 0.001, and with idealistic attitudes toward the police as trustworthy and prosocial *r*(1,314) = 0.52, *p* < 0.001. Conservatism also negatively correlated with idealistic attitudes toward the BLM protesters as trustworthy and prosocial, *r*(1,314) = −0.63, *p* < 0.001. Thus, political orientation in Study 2 appears again to have captured overall perceptions of the BLM movement (parallel analyses using the police and BLM-attitude measures in place of political orientation produce closely comparable patterns of association; see electronic supplementary material). As in Study 1, follow-up tests did not reveal any interaction between political orientation and video condition on funding preference, *p*s = 0.73–0.98.

### 10.2. Political orientation and state elevation

As in Study 1, the political orientation of the overall sample was somewhat left-leaning, *M* = −0.20, s.d. = 0.40, and an ANOVA revealed that state elevation differed across video conditions ($\eta_{p}^{2}=0.19$, *F*_2,1313_ = 156.30, *p* < 0.001). Pairwise comparison (figure 1) revealed that elevation was greater in the BLM video condition (*M* = 1.37, s.d. = 0.92) than in either the Control condition *(M* = 0.39, s.d. = 0.61, *p* < 0.001, 95% CI = [0.84, 1.10]), or the BtB condition (*M* = 0.91, s.d. = 0.90, *p* < 0.001, 95% CI = [0.32, 0.58]). State elevation was also greater in the BtB video condition than in the Control condition, *p* < 0.001, 95% CI = [0.39, 0.65].

**Figure 4.**
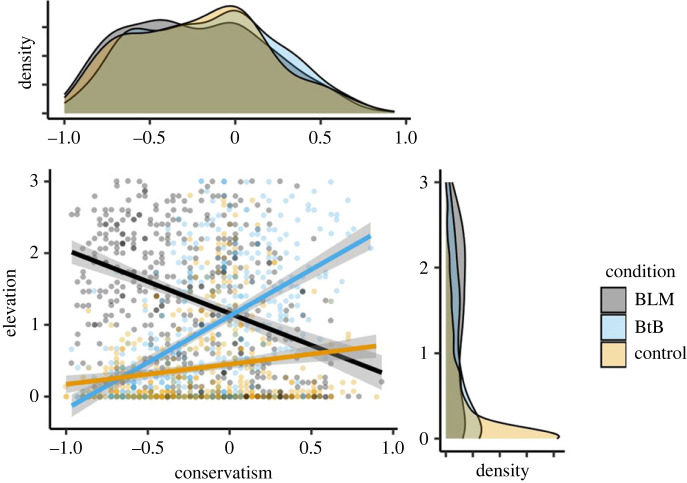
The association between political orientation and state elevation by video condition in Study 2. BLM = Black Lives Matter; BtB = Back the Blue.

A moderated linear regression model including state elevation as the dependent variable, political orientation as the independent variable and video condition as the potential moderator revealed significant interactions, *p*s < 0.001, in the associations between political orientation and elevation when contrasting the BLM and BtB conditions, *b* = −2.18, s.e. = 0.12, *t* = −17.62, the BLM and Control conditions, *b* = −1.17, s.e. = 0.12, *t* = −9.53, and the BtB and Control conditions, *b* = 1.02, s.e. = 0.12, *t* = 8.23. Consistent with Prediction 1, simple slopes analyses confirmed that conservative political attitudes were negatively associated with elevation in the BLM video condition, *b* = −0.89, s.e. = 0.09, *t* = −10.20, *p* < 0.001, 95% CI = [−1.06, −0.72], but positively correlated with elevation in the BtB video condition, *b* = 1.30, s.e. = 0.09, *t* = 14.70, *p* < 0.001, 95% CI = [1.12, 1.47] (figure 4). Against expectations, but as observed in Study 1, an effect of political orientation was again evident in the Control video condition, such that more conservative attitudes predicted greater elevation, *b* = 0.28, s.e. = 0.09, *t* = 3.30, *p* < 0.001, 95% CI = [0.11, 0.45]. (See electronic supplementary material, table S3 for comparable associations using non-parametric Spearman rank-order tests conducted given left-skewed ratings of elevation in the control condition.)

### 10.3. Partisan elevation and police funding preferences

An ANOVA found differences across conditions in funding preferences ($\eta_{p}^{2}=0.006$, *F*_2,1312_ = 3.88, *p* = 0.021). Pairwise comparisons revealed that participants favoured decreasing the percentage of current police funding in the BLM video condition, *M* = −4.31, s.d. = 44.59, to a greater extent than in the Control condition, *M* = 2.93, s.d. = 44.75, *p* = 0.044, 95% CI = [−14.31, −0.17], comparably to the trend observed in Study 1. There was also a significant difference between police funding preferences in the BLM video condition relative to the BtB condition (*M* = 3.09, s.d. = 44.59), *p* = 0.040, 95% CI = [−14.53, −0.27], with no difference in police funding preferences between the Control condition and the BtB video condition, *p* = 0.998 (see electronic supplementary material, figure S6).

**Figure 5.**
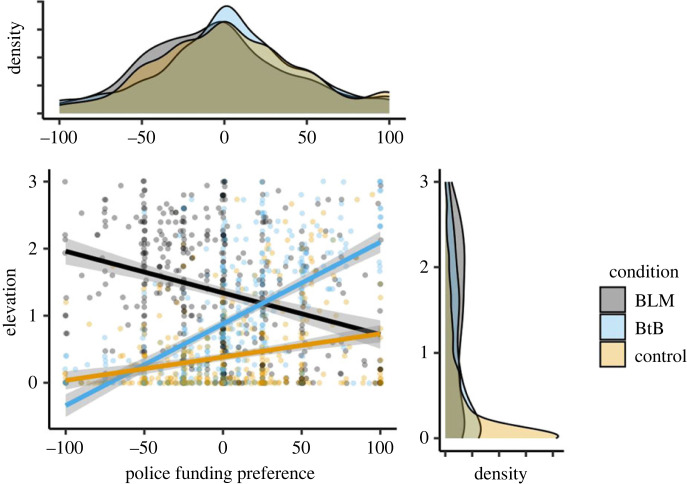
The association between police funding preferences and state elevation by video condition in Study 2. BLM = Black Lives Matter; BtB = Back the Blue.

We next tested the association between state elevation elicited by the video manipulation and police funding preferences. A moderated linear regression model including police funding preferences as the dependent variable, state elevation as the independent variable and video condition as the potential moderator revealed significant interactions, *p*s < 0.001, in the associations between elevation and funding preferences when contrasting the BLM and BtB conditions, *b* = −44.77, s.e. = 3.03, *t* = −14.76, the BLM and Control conditions, *b* = −33.82, s.e. = 3.80, *t* = −8.89, and the BtB and Control conditions, *b* = 10.95, s.e. = 3.82, *t* = 2.87. Consistent with Prediction 2, state elevation in the BLM video condition-tracked preferences to defund the police, *b* = −14.70, s.e. = 2.13, *t* = −6.92, *p* < 0.001, 95% CI = [−18.90, −10.60], whereas state elevation in the BtB video condition-tracked preferences to increase police funding, *b* = 30.00, s.e. = 2.16, *t* = 13.91, *p* < 0.001, 95% CI = [25.80, 34.30] (figure 5). In the Control video condition, as in Study 1, state elevation once again associated with preferences to increase funding to the police, *b* = 19.10, s.e. = 3.15, *t* = 6.06, *p* < 0.001, 95% CI = [12.90, 25.30]. (See electronic supplementary material, table S3 for comparable associations using non-parametric Spearman rank-order tests conducted given left-skewed ratings of elevation in the control condition.)

## 11. Discussion

In Study 2, we closely replicated our prior findings that political attitudes moderate feelings of elevation in response to the BLM video, and that state elevation elicited by the BLM video tracks preferences to defund the police. Further bolstering the hypothesis that support versus opposition to the BLM movement would determine state elevation, conservatism predicted greater elevation in response to the BtB video and elevation aroused by the BtB video correlated with preferences to increase police funding. Unexpectedly, despite the use of an alternate video intended to avoid political or affective connotations, as in Study 1, conservatism also mildly positively correlated with state elevation in the control condition, arguably making the inverse correlation in the BLM condition somewhat more striking. However, given the notably low levels of elevation in the control condition, this pattern merely suggests that conservatives are disposed to experience a slight degree of positive affect when viewing individuals engaged in productive tasks (e.g. repairing a broken computer).

In a departure from the trend observed in Study 1, police funding preferences were significantly lower in the BLM video condition relative to the control condition. However, this finding should be treated with caution, as (i) the significance level was marginal (*p* = 0.044) despite use of a relatively large sample, (ii) the contrast was non-significant in the previous study and (iii) there was no comparable funding preference shift in the BtB condition. Thus, although there do appear to be indications in both studies that exposure to the BLM video may have mildly inclined left-leaning participants to favour the movement's call to decrease police funding, funding preference ratings appear to have primarily reflected stable coalitional attitudes rather than outputs of state elevation.

## 12. General discussion

We tested whether prior attitudes toward a highly salient, politically divisive social conflict would modulate experiences of moral elevation. These studies provided an opportunity to assess whether the ASE model of elevation [16] would replicate using real-world stimuli. As predicted, we found in two studies that political orientation, used as an index of perceptions of the BLM movement as cooperatively aligned with, versus antagonistically opposed to, one's coalitional aims, determined whether participants experienced elevation in response to a video depicting large-scale protests demanding racial equity in policing. Further, in Study 2, we observed parallel effects with regard to the moral elevation experienced by conservatives in response to a BtB counter-protest video. In a complementary pattern, state elevation evoked by the BLM video-tracked preferences to defund the police, whereas state elevation evoked by the BtB video-tracked preferences to increase police funding, consistent with divergent left/right political attitudes regarding allocation of funds to policing versus other social services. To our knowledge, not only is this the first demonstration that state elevation can be elicited by political protests, but, more broadly, this is the first evidence that coalitional affiliation appears to be a key determinant of the capacity of prosocial coalitionary behaviour to elicit elevation in contexts of intergroup conflict.

Our online designs did not include measures of helping behaviour. Future inquiry into the coalitional dimensions of moral elevation should incorporate prosocial behavioural measures and explore the boundaries of prosociality in conflictual contexts. For example, we expect the state elevation experienced by partisans on either side of a given conflict to motivate altruistic behaviour of the sort documented in prior elevation research (e.g. charitable donations and time spent performing a helpful task), albeit primarily or exclusively directed toward the in-group. An important but unanswered question is whether elevation would similarly motivate altruistic acts of destruction or violence on behalf of one's coalition. On the one hand, conceptually, prosociality encompasses righteous punishment of those perceived to be wrongdoers or threats [65] and may be motivated by elevation, particularly in contexts in which engaging in virtuous punishment entails a significant cost to the punisher. On the other hand, the domain of prosocial outputs engendered by elevation may not encompass overtly harmful or destructive acts, which the ASE approach suggests might instead be motivated by other emotions related to coalitional antipathy such as anger, disgust or fear. Within the ASE model, the complex network comprised (i) attitudes toward relevant individuals or groups, (ii) scenarios in which individuals or groups are observed and (iii) links to diverse emotions which may be aroused by relevant scenarios, is termed a *sentiment* [10,66]. With regard to coalitional sentiments, an attitude such as out-group antipathy can theoretically potentiate or inhibit emotions such as elevation, anger, fear, pride and so forth in response to scenarios as they unfold. In this way, coalitional sentiments may parcel particular emotional responses to various scenarios in line with their functional specializations, such that elevation in response to cues of coalitional solidarity and shared sacrifice may give way to rage and aggressive tendencies in response to cues of transgression against the in-group. Future studies should explore the potential interplay of distinct emotions embedded within sentiments germane to coalitional conflict, and the scope of coalitional behaviours associated with the particular emotions aroused by distinct scenarios.

We observed striking interactions between coalitional attitudes and state elevation in response to stimuli related to a predominantly peaceful political conflict. Given the ubiquity of group conflicts of varying degrees of intensity over the course of human evolution, coalitional sentiments should be sensitive to the relative fitness stakes of conflict, suggesting that the interactions between group attitudes and state elevation observed in the present studies will be more pronounced in contexts of overtly violent intergroup conflict. For example, at the time of writing, the Russian Federation has invaded a sovereign nation, Ukraine, under a flimsy pretext of preemptive self-defense. Among members of nations antagonistic to Russia such as the United States, this has elicited outrage, donations of humanitarian aid and praise of Ukrainian acts of military resistance as heroically altruistic; indeed, a legion of foreign volunteer fighters is serving on the front lines [67]. The war thus provides abundant evidence of exceptional prosociality on the part of, and in solidarity with, the Ukrainian people. Correspondingly, stimuli depicting the efforts of Ukrainians to cooperate in defense of their country would presumably inspire elevation in U.S. samples, whereas the efforts of Russian soldiers working together to occupy Ukraine would not. By contrast, illustrative of the power of coalitional attitudes to shape scenario appraisals and subsequent emotions, the majority of U.S. Americans perceived their own country's invasion of the sovereign nation of Iraq, which was also committed under a flimsy pretext of preemptive self-defense, as morally laudable during the invasion, and were far from sympathetic with the efforts of Iraqis to resist the U.S.-led occupation of their country [68]. Such real-world parallels indicate that the coalitional dynamics of elevation that we have documented in the context of peaceful political demonstrations likely apply in contexts of active intergroup warfare and invite further study in the service of both basic research and potential translational efforts to ameliorate armed conflict.

Whereas our experiments probed the effects of witnessing cooperative protest behaviour dedicated to coalitional ends, the extent to which similar coalitional dynamics apply when observers witness non-coalitional acts of overt prosociality performed by individuals displaying markers of coalitional identity (e.g. a ‘Black Lives Matter’ or ‘Make America Great Again’ t-shirt) remains unclear. To the extent that an altruist's membership in one's own ingroup or an antagonistic out-group indexes the probability of cooperation beneficial to the observer, such markers of coalitional affiliation can be expected to moderate the capacity of their prosocial acts to elicit elevation. Further research might test whether coalitional affiliation comparably determines elevation (and related helping tendencies) even when the observed prosocial acts are unrelated to group conflict. Relatedly, for instance, Blomster Lyshol *et al*. [69] found that experimental primes depicting prosocial out-group individuals (as defined by race, nationality or sexual orientation) elicited an emotion similar to state elevation which, in turn, predicted reduced dehumanization and enhanced warmth toward these groups. Importantly, however, in these studies the out-group categories were not pertinent to active intergroup conflict, and the question remains whether depictions of such exemplars (e.g. at the time of writing: highly prosocial Russian soldiers helping one another) would comparably arouse elevation or attendant shifts in dehumanization or warmth.

Other studies assessing the impact of elevation-elicitation on group relations have used prosocial primes that are incidental to coalitional identification or active conflict. For example, Oliver *et al*. [70] found that participants exposed to videos depicting expressions of interpersonal warmth or coordination unrelated to group identity reported heightened state elevation, and elevation was associated with a greater sense of connectedness with members of racial out-groups (also see [71]). Although these results suggest a role for elevation in ameliorating racial prejudice, they are not relevant to circumstances of salient coalitional conflict. In research directly pertinent to active conflict, Shulman *et al*. [72] recently conducted three studies examining the potential for elevation to increase Israeli participants' support for humane policies toward the Palestinian people, finding that experimentally induced elevation did increase support for humanitarian policies (e.g. providing medical care to Palestinian children), but not political compromises (e.g. withdrawing from colonized territory). Our present findings suggest that, had Shulman *et al*.'s participants been assigned a video depicting large-scale, peaceful coordination among Palestinians demanding just treatment akin to the BLM video used here, the Israeli sample may not have evinced increased support for humanitarian policies toward Palestinians—and might instead have favoured less humane policies—to the extent that their baseline attitudes guided interpretation of the protest stimulus as coalitionally antagonistic. Although this prediction requires empirical testing, a translational lesson would obtain if confirmed: to enhance intergroup sympathy, eschew coalitional framing.

Our functionalist account of elevation as a mechanism that shapes costly behaviour so as to maximize payoffs for the actor predicts that, in coalitional contexts, elevation-elicitation will hinge on the personal relevance of the coalitions with respect to the likely benefits or risks of cooperating. Emerging work indicates that, even in quite small communities, there is substantial variation in attitudes concerning, and willingness to be prosocial toward, out-groups—variation that is largely explicable in terms of the positive and negative affordances of out-groups for each individual [73]. Crucially, however, if significant intergroup conflict erupts, cost-benefit calculations regarding cooperating within or across group lines necessarily change once other ingroup members adopt a with-us-or-against-us orientation that motivates them to more aggressively reward those who cooperate within the in-group and punish those who do not [35]. Accordingly, under circumstances of active coalitional antagonism, the capacity for elevation to be elicited can be expected to bifurcate more sharply along group lines, whereas cessation of active conflict can be expected to attenuate the relevance of group identity to elevation-elicitation. Future work might leverage real-world instances or experimentally contrived simulations of intergroup conflict versus accommodation to test these predictions.

Freeman *et al*. [74] found that prosocial stimuli modelling forgiveness and aid to out-groups despite their infliction of violent harms to in-group altruists increased the willingness of White participants to donate to a Black cause, whether or not the groups depicted in the prosocial manipulations were defined by race. Significant interactions were observed between these prosocial manipulations and individual differences in attitudes favouring group-based dominance (see [75]), such that group-based dominance was negatively correlated with donation amounts in the control condition, but not following the elevating examples of intergroup forgiveness. Future work might examine the impact of stimuli that depict prosocial intergroup cooperation but do not incorporate or reference violence or other harms, and/or which involve cooperation between groups of more direct relevance to the participants' own coalitional sentiments.

Elevation is similar to—perhaps even isomorphic with—a hypothesized emotion that researchers have labelled *kama muta*, the feeling of being ‘moved’ or ‘touched’ associated with the sudden intensification of a communal sharing relationship, that is, one in which the parties are equivalent in some key aspect of identity [76]. Kama muta is thought to be elicited by witnessing expressions of interpersonal closeness and is theorized to adaptively direct investment toward those relationships in which communal sharing is most likely to be profitable [77,78]. Analysing the content of social media materials designed to recruit support for social movements in the USA, Pierre [79] finds frequent use of themes and accounts apparently intended to elicit kama muta. Critically, the social movements examined constituted coalitional conflict (e.g. efforts in the USA to stop the planned Dakota Access Pipeline carrying fossil fuels across indigenous lands and sensitive ecosystems). Both our theory and our findings agree that emotions such as elevation / kama muta can be expected to track support for moralized coalitional struggle. Our approach adds the crucial qualifier that the extent to which the analysed materials elicited kama muta will have been contingent on the viewer's pre-existing coalitional attitudes. Departing from the kama muta model, we expect that stories of communal sharing in the service of coalitional aims do not possess an unqualified capacity to elicit emotions motivating increased prosociality, but rather have evocative power as a function of the prior coalitional alignment of their audience.

The present findings highlight both the promise of elevation to inspire cooperation in pursuit of political aims and the hazard of elevation as potentially escalating coalitional conflict in ways which exacerbate harm to all sides. Research indicates that upholding highly moralized coalitional objectives (i.e. ‘sacred values’) is viewed as an imperative duty, and therefore not subject to compromise, regardless of utilitarian cost-benefit considerations [80]. Elevation involves palpable experiences of being moved by acts imbued with moral rightness. Insofar as elevation enhances perceptions of one's in-group's struggle as morally righteous rather than as a means to obtain material concessions, experiences of elevation may undermine openness to compromise. If so, elevation may contribute to a social-emotional positive feedback loop that makes one side in a conflict increasingly unwilling to meet their opponents halfway: if this emotion is elicited by in-group members' displays of their willingness to incur costs rather than compromise, then in-group observers, energized by their experience of elevation, may harden their own stances in this regard, leading them to act in kind, thus creating a cascade which moves the in-group farther and farther away from the possibility of resolving conflict through negotiation.

## 13. Conclusion

In both scholarly literature and popular accounts, elevation and other positive emotions are sometimes presented as if they were a panacea for healing social divisions—as though human nature were designed such that mere exposure to selfless deeds would necessarily evoke recognition of our shared affinity. However, from a functional perspective, emotion adaptations such as elevation evolved to promote individual fitness, and relative fitness payoffs predict when and how emotions are elicited. In situations of intergroup conflict, prosocial behaviour in the service of coalitional goals evokes elevation in observers who side with those prosocial actors, but not in those who align with the opposing side. Actions that inspire elevation and altruistic self-sacrifice on the part of some observers will be met with cold indifference, or even hostility, contingent on representations of the affordances of individuals or groups vis-à-vis the observer's welfare.

## Data Availability

The datasets collected and analysed during the current studies are available on the Open Science Framework, https://osf.io/kdeg6/. The pre-registrations, full materials and datasets for the studies reported here are publicly archived (see https://osf.io/kdeg6/). The data are provided in the electronic supplementary material [81].
